# Clinical Features, Etiology and Outcomes of Community-Acquired Pneumonia in Patients with Chronic Obstructive Pulmonary Disease

**DOI:** 10.1371/journal.pone.0105854

**Published:** 2014-08-28

**Authors:** Joan Gómez-Junyent, Carolina Garcia-Vidal, Diego Viasus, Pere Millat-Martínez, Antonella Simonetti, Mª Salud Santos, Carmen Ardanuy, Jordi Dorca, Jordi Carratalà

**Affiliations:** 1 Department of Infectious Diseases, Hospital Universitari de Bellvitge, University of Barcelona, and Institut d’Investigació Biomèdica de Bellvitge (IDIBELL), Barcelona, Spain; 2 Department of Respiratory Medicine, Hospital Universitari de Bellvitge, University of Barcelona, and Institut d’Investigació Biomèdica de Bellvitge (IDIBELL), Barcelona, Spain; 3 Department of Microbiology, Hospital Universitari de Bellvitge, University of Barcelona, and Institut d’Investigació Biomèdica de Bellvitge (IDIBELL), Barcelona, Spain; 4 REIPI (Spanish Network for the Research in Infectious Diseases), Instituto de Salud Carlos III, Madrid, Spain; D’ or Institute of Research and Education, Brazil

## Abstract

**Background:**

Community-acquired pneumonia (CAP) is a frequent complication of chronic obstructive pulmonary disease (COPD), but previous studies are often contradictory.

**Objectives:**

We aimed to ascertain the characteristics and outcomes of CAP in patients with COPD as well as to determine the risk factors for mortality and *Pseudomonas aeruginosa* pneumonia in COPD patients with CAP. We also describe the etiology and outcomes of CAP in COPD patients receiving chronic oxygen therapy at home and those receiving inhaled steroids.

**Methods:**

An observational analysis of a prospective cohort of hospitalized adults with CAP (1995–2011) was performed.

**Results:**

We documented 4121 CAP episodes, of which 983 (23.9%) occurred in patients with COPD; the median FEV1 value was 50%, and 57.8% were classified as stage III or IV in the GOLD classification. Fifty-eight per cent of patients were receiving inhaled steroids, and 14.6% chronic oxygen therapy at home. Patients with COPD presented specific clinical features. *S. pneumoniae* was the leading causative organism overall, but *P. aeruginosa* was more frequent in COPD (3.4 vs. 0.5%; p<0.001). Independent risk factors for case-fatality rate in patients with COPD were multilobar pneumonia, *P. aeruginosa* pneumonia, and high-risk PSI classes. Prior pneumococcal vaccination was found to be protective. FEV_1_ was an independent risk factor for *P. aeruginosa* pneumonia.

**Conclusions:**

CAP in patients with COPD presents specific characteristics and risk factors for mortality. Prior pneumococcal vaccine has a beneficial effect on outcomes. *P. aeruginosa* pneumonia is associated with low FEV1 values and poor prognosis.

## Introduction

Chronic obstructive pulmonary disease (COPD) is one of the leading causes of morbidity and mortality worldwide. Recent projections predict that by 2030 it will be the fourth main cause of death and the seventh cause of the global burden of disease, presenting a significant increase compared to data from 2002 [1]. Its prevalence is around 1% across all age groups, increasing to 10% in patients aged 40 years and over. Around 2.5 million people die of the disease each year [2]–[5].

Community-acquired pneumonia (CAP) is one of the most frequent infections requiring hospitalization in developed countries [6]. In COPD patients, CAP is one of the most common infections [7]. Patients with COPD have structural disruptions in the lung parenchyma [8] and frequently receive antibiotic and oral or inhaled steroid treatment. Moreover, COPD is characterized by a chronic inflammation of the airways [9] and it has been suggested that patients may present changes in their local and systemic immune response [10]. For all these reasons, the presentation of CAP in patients with COPD may differ from that of patients without the condition. Prior studies have evaluated the characteristics of patients with COPD and CAP [11]–[20], but the results are contradictory: many issues such as factors related to mortality, risk factors for *Pseudomonas aeruginosa* pneumonia or etiology, and outcomes in patients with chronic oxygen therapy at home or using inhaled steroids are still unclear and in need of clarification.

The aims of this study were: 1) to determine the epidemiology, clinical features and outcomes of patients with COPD in a large prospective cohort of non-severely immunosuppressed hospitalized adults with pneumonia; 2) to analyse risk factors for mortality in patients with COPD and CAP; 3) to assess clinical characteristics, risk factors for and outcomes of *P. aeruginosa* pneumonia in patients with COPD, and 4) to describe the etiology and outcomes in patients with COPD receiving chronic oxygen therapy at home and in those receiving inhaled steroids treatment.

## Materials and Methods

### Ethics statement

The study was approved by the Ethical Committee of Hospital Universitari de Bellvitge. To protect personal privacy, identifying information of each patient in the electronic database was encrypted.

### Setting, patients and study design

This observational study was conducted at an 800-bed university hospital for adults in Barcelona, Spain. All non-severely immunosuppressed adult patients admitted to the hospital with pneumonia through the emergency department from February 1995 through October 2011 were prospectively recruited and followed up. Patients with neutropenia, solid organ transplantation, chemotherapy, acquired immunodeficiency syndrome or chronic corticosteroid therapy (≥20 mg prednisone/day or equivalent for at least two previous months) at admission were excluded.

### Clinical assessment and antibiotic therapy

Patients were seen daily during their hospital stay by one or more of the investigators, who recorded clinical data in a computer-assisted protocol. Data were collected on demographic characteristics, comorbidities, causative organisms, antibiotic susceptibilities, biochemical analysis, empirical antibiotic therapy, and outcomes.

At the initial visit, before starting empirical antibiotic therapy, patients underwent a complete clinical history and physical examination. Basic chemistry and hematology tests, arterial blood gas determinations and chest radiography were performed. Two sets of blood samples were obtained and cultured and, when available, a sputum sample was evaluated by Gram staining and culture. Urinary antigen detection tests for *Streptococcus pneumoniae* and *Legionella pneumophila* were performed if indicated by the attending physician.

Forced expiratory volume in the first second (FEV_1_), chronic oxygen therapy at home and inhaled therapy information were recorded after reviewing the spirometry and hospital databases. To stratify patients into pneumonia risk classes, the pneumonia severity index (PSI) was used [21].

Antibiotic therapy was initiated in the emergency department in accordance with the hospital guidelines, which recommend the administration of a β-lactam (ceftriaxone sodium or amoxicillin/clavulanate potassium) with or without levofloxacin. Combination therapy was recommended for patients with clinical suspicion of a *Legionella* species or an atypical pathogen, or in the absence of a demonstrative finding on sputum Gram stain results. Levofloxacin monotherapy was recommended for patients with a positive urine antigen test for *L. pneumophila* serogroup 1, as described elsewhere. Combined amoxicillin/clavulanate was recommended for patients with clinical suspicion of aspiration pneumonia.

### Definitions

Pneumonia was defined as the presence of an infiltrate on a chest radiography plus an acute illness associated with one or more of the following signs and symptoms: new cough with or without sputum production, pleuritic chest pain, dyspnea, fever or hypothermia, altered breath sounds on auscultation or leukocytosis [6].

COPD was defined as the coexistence of chronic and progressive symptoms such as dyspnea, cough and sputum and airflow obstruction diagnosed by spirometry (basal forced spirometry showing FEV_1_≤70% of its reference value and β_2_-agonist reversibility of predicted FEV_1_ of <15% and/or 200 ml. with FEV1/FVC <70%). Patients were stratified into four stages according to the Global Initiative for Chronic Obstructive Lung Disease (GOLD) criteria [22]. GOLD stage I or mild COPD was defined as FEV_1_≥80% predicted, GOLD stage II or moderate COPD as 50%≤ FEV_1_<80% predicted, GOLD stage III or severe COPD as 30% ≤FEV_1_<50% predicted and GOLD stage IV or very severe COPD as FEV_1_<30% predicted or FEV_1_<50% predicted with chronic respiratory failure.

Patients who had smoked more than 10 cigarettes per day for at least one year preceding the study were classified as current smokers. Alcohol abuse was considered if alcohol intake was >3 standard drinks per day. Pre-hospital antibiotic treatment was defined as the oral intake of antibiotic drugs >24 hours prior to hospitalization for the same episode of acute disease. Patients were classified as receiving antibiotics if they self-reported prescription of any of these medications or by reviewing the prescriptions from their General Practitioner at the SAP Healthcare Database of the Catalan Health Service (Institut Català de la Salut). Prior antibiotic treatment was defined as the intake of antibiotic drugs >3 months prior to hospitalization.

Vaccination status was assessed from interviews with the patients or their relatives and from reviews of hospital and personal health records (vaccination card). Patients were considered to be pneumococcal-vaccinated if the 23-valent polysaccharide pneumococcal vaccine had been administered in the five years before admission, and influenza-vaccinated if seasonal influenza vaccine had been administered during the year prior to admission. Patients were considered to be on chronic oral steroids if they received more than 5 mg prednisone/day or equivalent for at least the two previous months. Respiratory failure was defined as a PaO2/FiO2 ratio less than 300. The diagnosis of septic shock was based on a systolic blood pressure of less than 90 mmHg, and diagnosis of peripheral hypoperfusion on the need for vasopressors. A comorbid condition was defined as the presence of one or more disorders in addition to COPD. Initial inappropriate therapy was defined as the absence of antimicrobial agents directed at a specific type of organism or administration of an antibiotic to which the organism was resistant, according to susceptibility test criteria for lower respiratory tract pathogens.

Complications were defined as any untoward circumstances occurring during hospitalization, with the exception of side effects of medication. In-hospital cardiac events included acute coronary syndromes, arrhythmias and decompensated heart failure. Time to clinical stability was defined as previously described by Halm et al [23]. Early case-fatality rate was defined as death due to any cause <48 h after hospitalization. Overall case-fatality rate was defined as death due to any cause <30 days after hospitalization.

### Microbiological studies and etiologic diagnosis

Pathogens in blood, normally sterile fluids, sputum, and other samples were investigated using standard microbiological procedures. Sputum specimens were usually collected under the supervision of a registrar or nurse before antibiotic therapy was begun. Specimens were sent to the laboratory and processed immediately. No special procedures were performed for sputum samples if they could not be obtained spontaneously. A Gram stain was performed on a purulent portion of each sputum specimen and examined by trained personnel. The slides were evaluated for quality under low power (×10). Salivary contamination was defined by detecting squamous epithelial cells, and purulence was determined by the presence of polymorphonuclear cells. Sputum samples were considered of good quality if they had <10 squamous cells and >25 leukocytes per low-power field. Otherwise, the sputum sample was considered contaminated by saliva and rejected. Good-quality specimens were then screened for a predominant bacterial morphological type at oil immersion field (×100). A predominant morphotype was defined as the presence of a single morphotype that accounted for >75% of the organisms seen. Sputum cultures were processed immediately in blood agar, chocolate agar, and MacConkey agar media. Isolation of *Legionella pneumophila* was also attempted by use of buffered charcoal-yeast extract medium in selected cases.

The *Streptococcus pneumoniae* antigen in urine was detected by using a rapid immunochromatographic assay (NOW Assay; Binax Inc, Portland, Maine). *Legionella pneumophila* serogroup 1 antigen in urine was detected by an immunochromatographic method (NOW *Legionella* Urinary Antigen Test; Binax Inc) or enzyme-linked immunosorbent assay (ELISA-Bartels, Bartels, Trinity Biotech, Wicklow, Ireland). Standard serologic methods were used to determine antibodies against atypical agents (on admission and 3–4 weeks thereafter). Antimicrobial susceptibility was tested by the microdilution method, following the Clinical Laboratory Standard Institute methods and criteria. Presumptive aspiration pneumonia was diagnosed on a clinical and radiological basis in patients who had risk factors such us compromised consciousness, altered gag reflex, dysphagia, severe periodontal disease, putrid sputum, and radiographic evidence of involment of a dependent pulmonary segment or necrotizing pneumonia.

### Statistical analysis

Categorical variables were described using counts and percentages from available data. Continuous variables were expressed as the mean and standard deviation (SD) or median and interquartile range (IQR) for abnormally distributed data (Kolmogorov-Smirnov test).

To detect differences we used the chi-square test or Fisher exact test for categorical variables and *t* test or Mann-Whitney test for continuous variables, when appropriate. A multivariate analysis was carried out to determine independent risk factors for mortality in patients with COPD and in those with both COPD and *Pseudomonas aeruginosa* pneumonia. Significant variables detected in the univariate analysis and considered clinically important were entered into the multivariate analysis. The relative risks were expressed as odds ratios (OR) and 95% confidence intervals (CI). The goodness-of-fit of the model was evaluated by the Hosmer-Lemeshow test.

The results were analyzed using SPSS, version 15.0 (SPSS Inc, Chicago, Illinois). A P-value of <0.05 was considered statistically significant. All reported P-values are two-tailed.

## Results

### Patient characteristics

During the study period, we documented 4121 CAP episodes, of which 983 (23.9%) cases occurred in patients with COPD; the median FEV1 value was 50% and 57.8% of these patients were classified into stages III and IV of the GOLD classification. Fifty-eight per cent of patients were receiving inhaled steroids and 14.6% chronic oxygen therapy at home.

**Table 1** shows the demographic, clinical and laboratory findings of patients with and without COPD. Patients with COPD were older, more often male, current and ex-smokers and heavy alcohol consumers. They were also more likely to have other chronic comorbid conditions, especially chronic heart diseases and neoplastic diseases, but less likely to have dementia. They had received oral corticoids and previous antibiotics and had been vaccinated against pneumococcus and influenza more often than patients without COPD. Patients with COPD were also more frequently classified as high-risk PSI.

**Table 1:** Characteristics of pneumonia by study group.

Variable	Patients with COPD [n = 983],n (%)	Patients without COPD [n = 3138],n (%)	p value
**Demographic data**			
Age, median (IQR), years	74 (67–80)	67 (52–78)	<0.001
>65 years old	785 (79.9)	1730 (55.2)	<0.001
Male sex	890 (90.5)	1912 (61)	<0.001
Current smoker	252 (25.8)	827 (26.6)	<0.001
Ex-smoker	535 (54.8)	744 (23.9)	
Alcohol abuse	195 (20)	491 (15.8)	0.002
Influenza vaccine (season)	627 (71.4)	1199 (42.6)	<0.001
Pneumococcal vaccine, 5 years	226 (26.8)	381 (13.9)	<0.001
**Comorbid conditions**			
Diabetes mellitus	216 (22)	639 (20.4)	0.280
Chronic heart disease	265 (27)	688 (21.9)	0.001
Neoplastic disease	112 (11.4)	255 (8.1)	0.003
Chronic renal disease	67 (6.8)	219 (7)	0.943
Chronic liver disease	63 (6.4)	200 (6.4)	1
Cerebrovascular disease	69 (7)	243 (7.7)	0.490
Cognitive deficit (dementia)	38 (3.9)	174 (5.5)	0.038
Statin use^1^	89 (25.8)	220 (20.9)	0.062
Chronic oral steroids	102 (10.4)	159 (5.1)	<0.001
Pre-hospital antibiotics	191 (19.1)	729 (23.9)	0.002
Prior antibiotic treatment	227 (23.1)	443 (14.1)	<0.001
Prior hospitalization (90 days)^2^	91 (12.3)	129 (5.5)	<0.001
Criteria of health-care associated pneumonia^3^	194 (26.2)	461 (19.7)	<0.001
**Clinical features at presentation**			
Fever (≥38.0°C)	437 (45)	1645 (52.9)	<0.001
Acute onset of disease	446 (45.5)	1385 (44.3)	0.532
Days of clinical symptoms at admission (IQR)	3 (1–5)	4 (2–6)	<0.001
Cough	859 (87.6)	2592 (83.2)	0.001
Expectoration	734 (74.8)	1812 (58)	<0.001
Purulent sputum	550 (63.7)	1354 (54.9)	<0.001
Dyspnea	765 (78)	1794 (57.4)	<0.001
Tachypnea (≥30 breaths·min^−1^)	429 (48.5)	1229 (43.3)	0.006
Pleuritic chest pain	419 (42.7)	1276 (40.9)	0.334
Impaired consciousness	135 (13.8)	466 (14.9)	0.407
Septic shock	67 (6.8)	238 (7.6)	0.443
Diarrhea, vomiting	85 (8.7)	519 (16.6)	<0.001
Headache	96 (9.8)	575 (18.4)	<0.001
Arthromyalgias	139 (14.2)	693 (22.2)	<0.001
**Laboratory and radiographic findings at presentation**			
Respiratory failure (PaO2/FiO2<300 or PaO2<60 mmHg)	624 (76.2)	1563 (66.1)	<0.001
Multilobar pneumonia	255 (26)	1095 (75.3)	<0.001
Pleural effusion	134 (13.7)	558 (17.9)	0.002
Empyema	21 (2.1)	150 (4.8)	<0.001
Bacteremia	86 (9.1)	407 (13.3)	0.001
*High-risk PSI classes^4^*	720 (73.5)	1691 (54.1)	<0.001

COPD, chronic obstructive pulmonary disease; IQR, interquartile range; PSI, pneumonia severity index.

1 Data available only for 1396 patients.^ 2^Data available from January 2001, only for 3084 patients; ^3^Data available from January 2001, only for 3084 patients^ 4^Patients were stratified into the following risk classes according to the PSI score: low risk (≤90 points, classes I, II, and III) and high risk (>90 points, classes IV and V).

Regarding clinical features, patients with COPD were more likely to have cough, expectoration, purulent sputum, dyspnea, tachypnea, and respiratory failure at admission. Conversely, fever, diarrhea, headache, arthromyalgias, multilobar infiltrates, pleural effusion, empyema and bacteremia were less common. Moreover, patients with COPD were admitted to the hospital with fewer days of clinical symptoms than those without the condition.

### Causative organisms

**Table 2** shows the distribution of causative organisms in the two groups. *Streptococcus pneumoniae* was the most frequent causative organism in patients from both groups. *Haemophilus influenzae* and Gram-negative bacilli, especially *Pseudomonas aeruginosa*, were more frequent in patients with COPD, while *Legionella pneumophila*, atypical agents and aspirative pneumonia were less common. Excluding ICU patients, PA was the cause of pneumonia in 42 of 3727 patients (1.1%), 3.1% in COPD patients and 0.5% in non-COPD patients (p<0.001). In patients admitted in ICU, PA was the cause of pneumonia in 7 of 381 patients (1.8%), 7.1% in COPD patients and 0.6% in non-COPD patients (p<0.001).

**Table 2:** Etiology of pneumonia by study group.

Variable	Patients with COPD [n = 983], n (%)	Patients without COPD [n = 3138], n (%)	p value
*Streptococcus pneumoniae*	359 (36.5)	1047 (33.4)	0.069
Pneumococcal bacteremia	65 (6.9)	345 (11.3)	<0.001
*Haemophilus influenzae*	97 (9.9)	117 (3.7)	<0.001
Aspiration pneumonia	49 (5)	248 (7.9)	0.002
*Legionella pneumophila*	21 (2.1)	204 (6.5)	<0.001
Gram-negative bacilli	40 (4.1)	37 (1.2)	<0.001
*Pseudomonas aeruginosa*	33 (3.4)	16 (0.5)	<0.001
*Pseudomonas aeruginosa* bacteremia	10 (1.1)	4 (0.1)	<0.001
Atypical agents	15 (1.8)	152 (4.8)	<0.001
Others	29 (3)	178 (5.7)	0.001
No pathogen identified	412 (41.9)	1257 (40.1)	0.318

COPD, chronic obstructive pulmonary disease; 72 (7.3%) patients with COPD and 118 (3.7%) patients without COPD had more than one cause of community-acquired pneumonia.

Bacteremia occurred less often in patients with COPD than in those without (9.1% vs. 13.3%; p = 0.001), as did pneumococcal bacteremia. However, bacteremia caused by *Pseudomonas aeruginosa* was more common.

### Clinical outcomes

The susceptibility test for pathogen isolates indicated that most patients had received adequate initial empirical antibiotic therapy (**Table 3**). Patients with COPD had more ischemic cardiac events, and CAP was more frequently recurrent.

**Table 3:** Antibiotic therapy and outcomes of pneumonia by study group.

Variable	Patients with COPD [n = 983], n (%)	Patients without COPD [n = 3138], n (%)	p value
Inappropriate empiric antibiotic therapy	45 (7.8)	123 (6.9)	0.339
In-hospital complications	274 (27.9)	969 (31)	0.073
Cardiac complications	73 (7.4)	262 (8.4)	0.385
Ischemic cardiac complications	13 (1.3)	18 (0.6)	0.018
Recurrent CAP	40 (4.6)	61 (2.1)	<0.001
ICU admission	70 (7.1)	311 (9.9)	0.08
Need for mechanical ventilation	44 (4.5)	182 (5.9)	0.127
Length of intravenous therapy, median (IQR), days	4 (2–7)	4 (3–7)	0.041
Time to clinical stability, median (IQR), days	4 (2–8)	4 (2–7.25)	0.956
Length of hospital stay, median (IQR), days	8 (6–13)	8 (6–12)	0.004
Early case-fatality rate, ≤48 hours	14 (1.4)	75 (2.4)	0.069
Overall case-fatality rate	72 (7.3)	241 (7.7)	0.783

CAP: Community-acquired pneumonia; COPD, chronic obstructive pulmonary disease; ICU: Intensive care unit; IQR, interquartile range.

Patients with COPD had a longer intravenous therapy and length of hospital stay, although time to clinical stability was similar in the two groups. No differences in early and overall case-fatality rate were found between the groups. The overall case-fatality rate in patients hospitalized in conventional wards was 5.5% in COPD patients and 5.6% in non-COPD patients (p = 0.917). Analyzing only ICU patients, overall case-fatality rate was 25.7%, 21 of 70 (30%) in COPD patients and 77 of 311 (24.8%) in non-COPD patients (p = 0.330).

### Risk factors for case-fatality rates among patients with COPD

**Table 4** compares the demographic and clinical features of patients with COPD who died and in those who survived. Patients who died were older and more often presented use of chronic oral steroids, chronic renal disease, dementia, multilobar pneumonia, bacteremia and septic shock at hospital admission. They were also more likely to be classified into high-risk PSI classes, more commonly received inappropriate empirical treatment, and more frequently required intensive care unit (ICU) admission and mechanical ventilation. Pneumonia due to Gram-negative bacilli and aspiration pneumonia were significantly more common in these patients, as were cardiac, metabolic, acute renal complications and nosocomial infections.

**Table 4:** Factors associated with mortality in patients with COPD and pneumonia: univariate analysis.

Variable	Patients with COPD who died [n = 72], n (%)	Patients with COPD and alive [n = 911], n (%)	p value
**Demographic data**			
Age, median (IQR), years	80 (73–85)	74 (66–80)	<0.001
>65 years old	65 (91.5)	719 (79)	0.011
Male sex	64 (88.9)	825 (90.7)	0.621
Current smoker	15 (21.4)	236 (26)	0.477
Influenza vaccine (season)	35 (64.8)	592 (71.8)	0.268
Pneumococcal vaccine, 5 years^1^	8 (15.4)	218 (27.6)	0.054
Chronic oral steroids	16 (22.9)	86 (9.5)	<0.001
Statin use	4 (17.4)	85 (26.4)	0.340
**COPD data**			
COPD stages III or IV	8 (42.1)	158 (29)	0.220
Chronic oxygen therapy	4 (10.5)	104 (14.9)	0.462
Inhaled steroids	21 (56.8)	401 (58.3)	0.854
FEV1, median (IQR), %	41 (31.5–57.5)	49 (36–62)	0.279
**Comorbid conditions**			
Diabetes mellitus	14 (19.4)	202 (22.2)	0.587
Chronic heart disease	25 (34.7)	239 (26.3)	0.119
Neoplastic disease	7 (9.7)	105 (11.5)	0.641
Chronic renal disease	12 (16.7)	55 (6)	0.001
Dementia	8 (11)	30 (3.3)	0.001
**Clinical features at presentation**			
Septic shock	14 (19.4)	53 (5.8)	<0.001
Multilobar pneumonia	32 (45.1)	223 (24.6)	<0.001
Bacteremia	14 (20.6)	71 (8.1)	0.001
High-risk PSI classes n (%)^2^	69 (95.8)	650 (71.7)	<0.001
**Etiology**			
*Streptococcus pneumoniae*	21 (29.2)	337 (37)	0.182
*Legionella pneumophila*	1 (1.4)	20 (2.2)	0.648
*Haemophilus influenzae*	7 (9.7)	90 (9.9)	0.963
Aspiration pneumonia	7 (9.7)	42 (4.6)	0.055
Gram-negative bacilli	14 (19.4)	24 (2.6)	<0.001
*Pseudomonas aeruginosa*	13 (18.1)	20 (2.2)	<0.001
Atypical agents	2 (2.8)	16 (1.8)	0.535
Other organisms	2 (2.8)	27 (3)	0.927
**Antibiotic therapy and outcomes**			
Inappropriate empirical treatment	10 (13.9)	33 (3.6)	0.01
Mechanical ventilation	17 (23.9)	26 (2.9)	<0.001
ICU admission	21 (29.6)	48 (5.3)	<0.001
Cardiac complications	18 (25.4)	55 (6)	<0.001
Acute renal complications	15 (21.1)	20 (2.2)	<0.001
Metabolic complications	7 (9.9)	21 (2.3)	<0.001
Nosocomial infection	4 (5.6)	16 (1.8)	0.026

DM, diabetes mellitus; FEV1: Forced expiratory volume in the first second; ICU: Intensive care unit; IQR, interquartile range; PSI, pneumonia severity index.

1 Data available only for 52 patients and 790 patients respectively; ^2^Patients were stratified into the following risk classes according to the PSI score: low risk (≤90 points, classes I, II, and III) and high risk (>90 points, classes IV and V).

The results of the multivariate logistic regression analysis for factors potentially associated with overall case-fatality rates in patients with COPD are summarized in **Table 5**. After adjustment, multilobar pneumonia, *Pseudomonas aeruginosa* pneumonia, and high-risk PSI classes were found to be independent risk factors for case-fatality rates. Conversely, pneumococcal vaccine had a protective effect. The goodness-of-fit of the model was 0.908.

**Table 5:** Factors associated with mortality in patients with COPD and pneumonia: multivariate analysis.

Variable	OR	95% confidence interval	p value
>65 years old	1.374	0.454–4.157	0.573
Chronic oral steroids	2.454	0.816–7.380	0.110
Chronic renal disease	2.211	0.573–8.531	0.249
Dementia	1.887	0.439–8.113	0.394
Multilobar pneumonia	2.883	1.299–6.399	0.009
Bacteremia	0.529	0.160–1.756	0.298
Septic shock	2.574	0.886–7.476	0.082
*Pseudomonas aeruginosa* pneumonia	19.091	4.326–84.256	<0.001
Inappropriate empirical treatment	2.334	0.711–7.657	0.162
Cardiac complications	2.594	0.824–8.172	0.103
High-risk PSI classes	10.316	1.691–62.946	0.011
Metabolic complications	1.349	0.243–7.500	0.732
Nosocomial infection	0.484	0.067–3.486	0.471
Pneumococcal vaccine	0.232	0.072–0.754	0.015

COPD, chronic obstructive pulmonary disease; OR: odds ratio; PSI, pneumonia severity index.

### Risk factors for Pseudomonas aeruginosa pneumonia in patients with COPD

We compared the characteristics of patients with COPD caused by *Pseudomonas aeruginosa* pneumonia and those with pneumonia caused by another pathogen (**Table 6**). Chronic liver disease and treatment with chronic oral steroids were more common in COPD patients with *Pseudomonas aeruginosa* pneumonia. These patients were also more likely to be classified as GOLD stages III and IV (50% versus 28.8%; p = 0.048), and to have received chronic oxygen therapy at home (30.8% versus 14%; p = 0.018), inhaled anticholinergics (80% versus 60.5%; p = 0.050) and long-acting beta-agonists (72% versus 51.1%; p = 0.040). Median (IQR) of FEV_1_ values was 40% (30–49) in patients with *Pseudomonas aeruginosa* pneumonia and 49% (36–72) in the others. No significant differences in PSI class were found between groups (63.6% versus 73.8%). Patients with *Pseudomonas aeruginosa* pneumonia more frequently received inappropriate empiric antibiotic therapy (42.4% versus 3.3%; p<0.001). Patients with COPD and *P.aeruginosa* pneumonia who received inappropriate empiric therapy were commonly classified as GOLD stage IV (57%) and frequently received inhaled steroids (70%). Only one patient was taking chronic oral steroids treatment. Early (15.2% versus 0.9%; p<0.005) and overall (39.4% versus 6.2%; p<0.001) case-fatality rates were higher.

**Table 6:** Characteristics of patients with COPD who had *Pseudomonas aeruginosa* pneumonia and those patients with COPD with pneumonia non-caused by *P. aeruginosa*.

Variable	Patients with *P. aeruginosa* pneumonia [n = 33], n (%)	Patients without *P. aeruginosa* pneumonia [n = 950], n (%)	p value
**Demographic data**			
Age, median (IQR), years	74 (68.25–77)	74 (67–80)	0.108
>65 years old	27 (84.4)	758 (79.8)	0.524
Male sex	31 (93.9)	859 (90.4)	0.497
Current smoker	7 (21.2)	245 (26.0)	0.066
Ex-smoker	24 (72.7)	511 (54.1)	0.066
Alcohol abuse	6 (18.2)	189 (20.1)	0.499
Influenza vaccine (season)	22 (81.5)	605 (71.1)	0.240
Pneumococcal vaccine, 5 years	11 (44)	215 (26.3)	0.049
**Comorbid conditions**			
Diabetes mellitus	9 (27.3)	207 (21.8)	0.455
Chronic heart disease	5 (15.2)	260 (27.4)	0.120
Neoplastic disease	4 (12.1)	108 (11.4)	0.894
Chronic renal disease	1 (3.0)	66 (6.9)	0.380
Chronic liver disease	5 (15.2)	58 (6.1)	0.037
Cerebrovascular disease	2 (6.1)	67 (7.1)	0.826
Cognitive deficit (dementia)	0 (0)	38 (4.0)	0.241
Statin use^1^	1 (5.6)	88 (26.9)	0.044
Chronic oral steroids	7 (21.9)	95 (10.0)	0.031
Pre-hospital antibiotics	6 (18.8)	175 (19.1)	0.958
Prior antibiotic treatment	10 (30.3)	217 (22.8)	0.342
Prior hospitalization^2^	4 (13.8)	87 (12.2)	0.802
**Clinical features at presentation**			
Fever (≥38.0°C)	7 (22.6)	430 (45.7)	0.011
Acute onset of disease	11 (33.3)	435 (45.9)	0.155
Cough	31 (93.9)	828 (87.3)	0.259
Expectoration	30 (90.9)	704 (74.3)	0.030
Purulent sputum	25 (83.3)	525 (63.0)	0.023
Dyspnea	29 (87.9)	736 (77.6)	0.163
Tachypnea (≥30 breaths·min^−1^)	14 (51.9)	415 (48.4)	0.726
Pleuritic chest pain	16 (48.5)	403 (42.5)	0.492
Impaired consciousness	4 (12.1)	131 (13.8)	0.781
Septic shock	4 (12.1)	63 (6.6)	0.219
Diarrhea, vomiting	2 (6.1)	83 (8.7)	0.590
Headache	5 (15.2)	91 (9.6)	0.290
Arthromyalgias	5 (15.2)	134 (14.1)	0.869
**Laboratory and radiographic findings at presentation**			
Respiratory failure (PaO2/FiO2<300 or PaO2<60 mmHg)	20 (76.9)	604 (76.2)	0.929
Multilobar pneumonia	9 (28.1)	246 (26.0)	0.074
Pleural effusion	3 (9.4)	131 (13.8)	0.471
Empyema	0 (0)	21 (2.2)	0.388
*High-risk PSI classes^3^*	21 (63.6)	699 (73.8)	0.193

COPD, chronic obstructive pulmonary disease; IQR, interquartile range; PSI, pneumonia severity index.

1 Data available only for 345 patients. ^2^Data available for 740 patients.^ 3^Patients were stratified into the following risk classes according to the PSI score: low risk (≤90 points, classes I, II, and III) and high risk (>90 points, classes IV and V).

After adjustment for chronic oral steroids, chronic liver disease, GOLD stages III or IV, chronic oxygen therapy at home, inhaled anticholinergics and inhaled long-acting beta-agonists, only the FEV1 value was found to be an independent risk factor for *Pseudomonas aeruginosa* pneumonia (OR 0.166, IC 95% 0.032–0.863, p = 0.028).

### Etiology and outcomes of patients with COPD who received chronic oxygen therapy at home and those with inhaled steroids

In a further analysis, etiology and outcomes of patients with COPD with and without chronic oxygen therapy at home were compared. Although *S. pneumoniae* was the most common causative organism in both groups (37% and 39.4% respectively), *Pseudomonas aeruginosa* was more frequent in patients with chronic oxygen therapy at home (7.4% versus 2.9%; p = 0.018) while aspirative pneumonia was less common (0% versus 4.4%; p = 0.026). No differences in early and overall case-fatality rates were found.

In the last analysis, we compared etiology and outcomes of patients with COPD who received inhaled steroids with those who did not. Pneumococcal pneumonia was the most common etiology in both groups (36.7% and 42.2% respectively). No differences in etiologies and early and overall case-fatality rates were found between groups.

## Discussion

This prospective study of a large cohort of patients offers a detailed evaluation of characteristics, etiology, and outcomes of pneumonia in patients with COPD. The main findings were that: 1) patients with and without COPD had different clinical features at admission; 2) although *S. pneumoniae* was the most frequent causative organism, *P. aeruginosa* pneumonia and *P. aeruginosa* bacteremia were more frequent in patients with COPD; 3) mortality rates were similar in patients with and without COPD; 4) independent risk factors for mortality in patients with COPD were multilobar pneumonia, *P. aeruginosa* pneumonia, and high-risk PSI classes; 5) pneumococcal vaccination was associated with better outcomes; 6) the only independent risk factor for *P. aeruginosa* pneumonia in patients with COPD was the FEV_1_ value; 7) no differences in mortality were found between patients with COPD who were receiving chronic oxygen therapy at home and those who were not; and 8) there were no differences in either etiology or outcomes between patients with COPD who were receiving inhaled steroids and those who were not.

We found COPD to be a frequent comorbid condition in patients with CAP (23.9%). These results are in agreement with those of other researchers who reported rates between 15% and 42%, depending on the population studied [12]–[14], [16]–[18], [20], [24]. Comparing the clinical picture of CAP at admission in patients with and without COPD, patients with COPD presented more often with cough, sputum and respiratory failure, as expected. Interestingly, fever, extensive radiographic infiltrates or pleural effusion/empyema were less frequent. This is consistent with data from Sellares and coworkers [19], who also documented a decreased incidence of parapneumonic pleural effusion among patients with chronic respiratory disorders treated with inhaled steroids. These findings may be associated with the higher use of previous antibiotics and steroids, and/or with the fact that patients with COPD seek clinical advice earlier. It has recently been shown that patients with COPD and CAP exhibit a different inflammatory response with lower levels of certain serum biomarkers; this finding may also help to explain these clinical differences [10].

The most frequent causative organism of pneumonia in all patient groups was *S. pneumoniae*. However, pneumococcal bacteremia was less frequent in patients with COPD. It is tempting to attribute this finding to the higher rates of pneumococcal vaccine recorded. Other possible explanations are the earlier time of consultation in patients with COPD or the fact that the pneumococcal serotypes isolated in COPD may differ from those found in other patients. Further information is needed regarding the microbiological characteristics of pneumococcal pneumonia in patients with COPD.

The association of *P. aeruginosa* and COPD is well known, but information regarding risk factors for *P. aeruginosa* pneumonia among the COPD population is scarce [12], [16]–[18], [24]. We found that patients with oxygen therapy at home presented more *P. aeruginosa* pneumonia, but the only independent risk factor for *P. aeruginosa* pneumonia in patients with COPD was FEV1 value. Previous studies have also reported that markers of respiratory functional impairment are the most important factors related to *P. aeruginosa* isolation in the setting of COPD exacerbation [25], [26]. Identifying patients with COPD with a higher risk of *P. aeruginosa* pneumonia seems mandatory, as we found that this population frequently received inappropriate antibiotic treatment (more than 40%), and have high early (15.2%) and late mortality (39.4%). Lack of coverage against *P. aeruginosa* has been associated with early mortality in patients with CAP [27]. Therefore, *P. aeruginosa* should be taken into consideration when selecting an initial empirical antibiotic treatment in hospitalized patients with CAP and severe COPD, especially in those receiving oxygen therapy at home and those admitted in ICU.

In agreement with previous reports [28]–[30], patients with COPD more frequently had recurrent CAP, ischemic cardiac complications, and prolonged length of hospital stay. However, early and overall mortality rates were similar in patients with and without COPD, as other authors have suggested [12], [20]. Although patients with COPD have poorer respiratory function, they are admitted to hospital sooner and have lower rates of multilobar pneumonia, empyema and bacteremia. This setting suggests a more balanced inflammatory response. The rates of early mortality are notably low in patients with COPD and come close to achieving statistical significance compared with patients without COPD.

We found that multilobar pneumonia, *Pseudomonas aeruginosa* pneumonia, and high-risk PSI class were associated with overall case-fatality rate in patients with COPD. These factors have been reported in previous studies [12], [14], [17], [27]. Recent studies have documented an increased incidence of acute cardiac events in patients with CAP and have associated these events with poor outcomes [28], [31]. In our study, patients with cardiac complications have higher mortality in the univariate analysis. Chronic oral steroids alter immune host response and exert a decisive influence on macrophage and granulocyte late function. As previously suggested [32], recovery of lung homeostasis could be seriously compromised by steroid administration at a late stage of infection. Although it has been suggested that inhaled steroids produce an excess risk of severe pneumonia in patients with COPD [33], the data regarding their impact on mortality are contradictory [34]–[36]. In our study, we did not find increases in early and overall mortality among patients receiving oral or inhaled steroids.

We found polysaccharide pneumococcal vaccination to be an independent protective factor against mortality in patients with COPD. Only 26.8% of patients with COPD had previously received pneumococcal vaccine, despite the fact that the US Advisory Committee on Immunization Practices recommends this practice for all patients with COPD [37]. Pneumococcal vaccination is believed to decrease the rates of invasive disease caused by *S. pneumoniae* [38]–[40]. Furthermore, previous reports have also found that pneumococcal vaccination may improve outcomes in patients with pneumonia [41], [42], including patients with chronic lung diseases [43]. These findings should convince clinicians of the importance of improving compliance with existing pneumococcal vaccination recommendations in patients with COPD.

The strengths of this study are the large number of patients included, its prospective design, and the comprehensive clinical and microbiologic data gathered. In addition, all patients with COPD included in the study had a spirometric confirmation of the disease. However, there are some limitations that should be acknowledged. Firstly, the study was conducted at a single center and the sample size was relatively small for some subsets of patients with COPD such as those who died. Secondly, some factors related with COPD function such as functional dependence, dyspnea score, walking test or BODE index were not available for the study. These factors may have influenced COPD mortality and/or risk for *P. aeruginosa* pneumonia. Finally, we lack information regarding prior isolation of *P. aeruginosa* in sputum in patients with COPD.

In conclusion, CAP in patients with and without COPD presents distinct clinical features. Although *S. pneumoniae* is the leading causative organism, Gram-negative bacilli and *P. aeruginosa* should also be taken into account, especially in patients with severe COPD receiving oxygen therapy at home. Multilobar pneumonia, *Pseudomonas aeruginosa* pneumonia, and high-risk PSI class were associated with overall case-fatality rate in patients with COPD. Conversely, prior pneumococcal vaccine was found to be protective, which should encourage physicians to increase the use of this vaccine in the COPD population.
